# Antimicrobial Effects of Basil, Summer Savory and Tarragon Lyophilized Extracts in Cold Storage Sausages

**DOI:** 10.3390/molecules26216678

**Published:** 2021-11-04

**Authors:** Artur Macari, Rodica Sturza, Ildiko Lung, Maria-Loredana Soran, Ocsana Opriş, Greta Balan, Aliona Ghendov-Mosanu, Dan Cristian Vodnar, Daniela Cojocari

**Affiliations:** 1 Faculty of Food Technology, Technical University of Moldova, 9/9 Studentilor Street, MD-2045 Chisinau, Moldova; artur.macari@tpa.utm.md (A.M.); aliona.mosanu@tpa.utm.md (A.G.-M.); 2 National Institute for Research and Development of Isotopic and Molecular Technologies, 400293 Cluj-Napoca, Romania; loredana.soran@itim-cj.ro (M.-L.S.); ocsana.opris@itim-cj.ro (O.O.); 3 Department of Preventive Medicine, “Nicolae Testemitanu State” University of Medicine and Pharmacy, 165 Stefan cel Mare Bd., MD-2004 Chisinau, Moldova; greta.balan@usmf.md (G.B.); daniela.cojocari@usmf.md (D.C.); 4 Faculty of Food Science and Technology, University of Agricultural Sciences and Veterinary Medicine, 3-5 Manăştur Street, 400372 Cluj-Napoca, Romania; dan.vodnar@usamvcluj.ro

**Keywords:** aromatic plants, extracts, antimicrobial activity, sausages, quality

## Abstract

The problem of functional foods with bioactive components of natural origin is current for the food industry. Plant extracts rich in polyphenols with antioxidant and antimicrobial activity are a promising source for use in improving the quality and characteristics of fresh meat and meat products. In this context, the purpose of the present study was to evaluate the physico-chemical, microbiological, sensory properties of sausages prepared with the addition of lyophilized extract of basil, thyme or tarragon. For the beginning, the total amount of polyphenols, the antioxidant and antimicrobial activity of the extracts obtained from three spices were evaluated. In the sausages previously infected with *Staphylococcus aureus* and *Escherichia coli* it was observed that there is a much larger number of colonies of microorganisms in the control sample compared to the other samples within 24 and 48 h. Moreover, following the addition of sausage extracts, no changes were found regarding their sensory acceptability.

## 1. Introduction

The functional foods are considered to be those foods that are intended to be consumed as part of the normal diet and that contain additional biologically active components that offer the potential for increased health or reduced risk of disease [1].

The interest for this category of food products has increased and the aim is to develop standards and guidelines for the development and promotion of such foods. Consumer interest in the relationship between food and health has grown substantially in Europe. There is a much broader recognition that today people can reduce the risk of disease and maintain their health and well-being through a healthy lifestyle, including diet.

The important role of foods such as fruits, vegetables, and whole grains in disease prevention, as well as the latest research on dietary antioxidants and combinations of plant protection substances, has provided an impulse for the development of the functional food market [2].

The use of plant extracts as a source of bioactive compounds is becoming an attractive strategy for improving the quality and characteristics of fresh meat and meat products [3]. Indeed, given their natural origins, bioactive compounds obtained from plants are ideal candidates to replace synthetic antioxidants (generally considered less safe) and to increase the shelf life of meat products. At the same time, these plant extracts can improve, either directly or indirectly, the functional value of meat products [4].

Antimicrobial compounds are used to inhibit the growth of microorganisms that induce food spoilage and antioxidants to retard lipid oxidation and discolouration of food [5,6]. Without food additives, adverse effects may be expected, such as more product recalls, an increased number of food-borne illness and subsequently increased the amount of food waste. Food producers therefore experience a severe limitation on the number of useful additives available. It is not possible to remove all preservatives without serious consequences on product safety and quality, but for the harmful effects of these compounds on humans, it is preferable to use the minimum quantities necessary for food preservation or changes with natural compounds. The challenge for clean label products is to find new food additives that meet both the food industry demand of having antioxidant and antimicrobial potency as well as consumer’s demand of being natural without compromising sustainability [7].

The meat industry is an extremely important food sector in European countries and provides a nutritionally dense food that contains a wide range of nutrients such as proteins, lipids, vitamins and minerals. The meat sector has faced years of frequent crisis concerning safety, quality and negative publicity. It is therefore important to contribute to an increase in the confidence for meat as a healthy food choice. The microbial spoilage of minced meat products is a heterogeneous process that involves the development of diverse and poorly characterised microbial communities. Despite the fact that bacterial growth is one of the main factors that makes meat objectionable for human consumption, less is known about their community dynamics.

Extracts from various fruit and plants are known to contain candidates for natural food additives with antioxidant and antimicrobial activity [8], and having positive effects on colour.

Recently, researchers from the University of Aarhus in Denmark and Danish Meat Research Institute proposed the use of herbs and berries in organic meat products, starting from the fact that some berries, leaves, bulbs, roots, and stems of some plants are known for their content in substances with antibacterial and antiviral properties. In some plants, the concentration of these compounds is so high that they can probably be used to preserve food [9].

The research started with 37 species of plants whose antibacterial properties were tested on *Listeria monocytogenes*, *Salmonella typhimurium* and *Escherichia coli*, the list being finally reduced to eight species with demonstrable conservation capacities: aronia, sage, savory, blackthorn, cranberries, wild garlic, red currant, and horseradish [3,10].

Each of them can be added in various combinations and quantities in meat products for preservation, and in most cases, they add pleasant and desired flavours to the meat products. The researchers also aimed to develop optimal processing and storage methods so that the desired properties are preserved as long as possible after harvesting. It has also been investigated how a homogeneous distribution of preservatives in meat can be obtained if they are to be mixed in powder form into the product which presents a liquid form (suspension) [10,11].

A solution in this sense is the lyophilization of plants or of plant extracts with biological value to obtain high quality products. The original shape of the product is maintained, and by rehydration, a product with an excellent quality can be obtained [12,13]. Moreover, it is an excellent method for preserving a wide variety of heat-sensitive materials, such as proteins, vitamins, essential oils, tannins, antioxidants, pharmaceuticals, tissues, and plasma [14,15].

The aim of this work was to study the addition effect of the lyophilized extract of basil, summer savory and tarragon on the physico-chemical quality indicators and microbiological activity of sausages. The spices chosen for this study were basil (*Ocimum basilicul* L.), summer savory (*Satureja hortensis* L.) and tarragon (*Artemisia dracunculus* L.)

## 2. Results

### 2.1. Plant Extracts Characterization

#### 2.1.1. Total Phenolic Content

**Figure 1:**
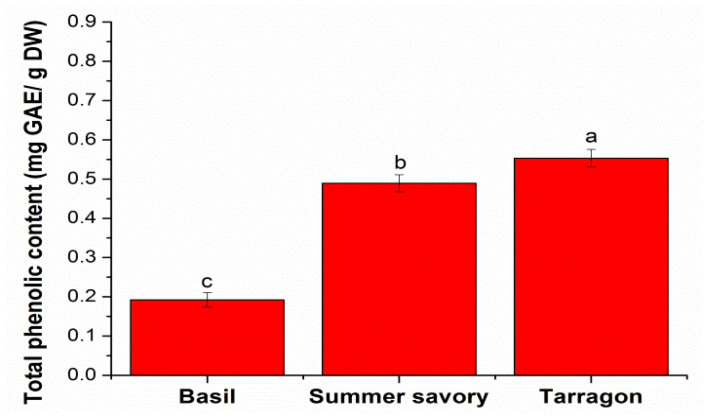
Comparative diagram of total phenolic content in the spices taken in study. Different letters (a, b, c) mean significant differences between the spices, determined by Tukey’s test (*p* < 0.05).

The total phenolic content of the analysed extracts was calculated using the calibration curve, performed under the same conditions as the sample solutions. For the calibration curve, the solutions were prepared by successive dilutions, in the range 0.001–0.070 mg/mL, from a standard solution of gallic acid with 1 mg/mL concentration. The equation of the calibration curve was y = 0.0133x + 0.0635, with a correlation coefficient of 0.9993. The results were expressed in mg gallic acid/g dry plant material. The total phenolic content obtained for basil and summer savory is presented in Figure 1.

The total phenolic content of the spices taken into account in this study varies between 0.19–0.55 mg GAE/g DW depending on the plant. Thus, the largest total phenolic content was obtained for tarragon extract, followed by summer savory extract, while for basil extract was obtained for the smallest amount of the three spices. The amount of polyphenols varies on the origin of the plant, as well as on the extraction conditions. Thus, in other studies depending on solvent extraction and used technique, the total phenolic content obtained for basil was 1.325 g/100 g DW with distilled water at 70 °C by infusion 15 min [16] and 516.35 mg/100 g DW with double-distilled water at 100 °C by infusion 15 min [17]. Total phenolic amounts between 63.2–109.6 mg GAE/100 g DW were obtained by extraction with 80% ethanol depending on the basil moisture [18]. Aburigal et al. [19] determined the total phenolic content obtained from basil plants were collected from a different region, by maceration in ethanol. They found it to vary from 0.408 to 0.881 mg GAE/g DW depending on the region where it comes from. Chan et al. [20] reported that 0.45 mg GAE/g DW was determined from extracts obtained by continuous shaking for 1 h at room temperature with methanol. Moreover, Słowianek and Leszczyńska [21] in the same condition, using 80% methanol, found 26.50 mg/g DW. For tarragon, Petkova et al. [22] found 25 mg GAE/g DW using ultrasound extraction and distilled water as solvent, while 58.03 mg GAE/g DW was obtained using methanol as extraction solvent for extraction on magnetic shaker [23]. For summer savory, other authors found 12.14 mg GAE/g DW using 60% aqueous ethanol as extraction solvent [24] and total phenolic amounts between 119.28–151.54 mg GAE/g DW depending on the used extraction technique and extraction solvent [25].

#### 2.1.2. Antioxidant Capacity

**Figure 2:**
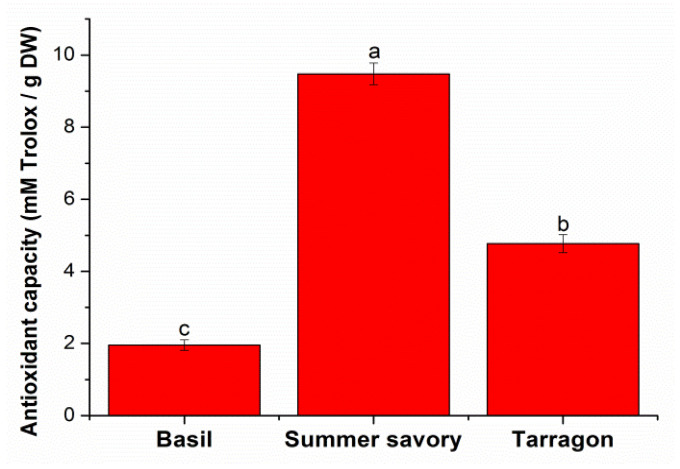
DPPH radical scavenging activity of studied spices. Different letters (a, b, c) mean significant differences between the spices, determined by Tukey’s test (*p* < 0.05).

The antioxidant capacity of the extracts was calculated from the calibration curve: y = 0.0016x + 0.0113, with a correlation coefficient of 0.9990. For this, the absorbances corresponding to solutions of different Trolox concentrations (0.004–3.2 mM) at a wavelength of 515 nm were read. Among the plants taken in this study (Figure 2), summer savory presented the highest antioxidant capacity (9.48 mM Trolox/g DW), followed by tarragon (4.77 mM Trolox/g DW) and then basil (1.96 mM Trolox/g DW).

#### 2.1.3. Antimicrobial Capacity

All investigated plant extracts showed significant bioactivity against the tested enterobacteria like *Listeria monocytogenes*, *Pseudomonas aeruginosa*, *Salmonella typhimurium*, and *Escherichia coli*, when compared with streptomycin as control (Table 1). Considering *Listeria monocytogenes*, both basil and tarragon extracts exerted similar inhibitory (MIC) and bactericidal (MBC) activities, recording concentrations of 1.25 mg/mL and 2.5 mg/mL, activities that were weaker than the antimicrobial activity of summer savory (0.31 mg/mL and 0.62 mg/mL). *Pseudomonas aeruginosa* were proved to be more sensitive to tarragon (MIC = 2.5 mg/mL, MBC = 5 mg/mL) than to basil and summer savory (MIC = 5 mg/mL, MBC = 10 mg/mL). All the three extracts proved an identical antimicrobial capacity towards *Salmonella typhimurium,* recording a MIC of 5 mg/mL and MBC of 10 mg/mL. *Escherichia coli* showed higher sensitivity to summer savory (MIC = 2.5 mg/mL, MBC = 5 mg/mL) than to basil and tarragon (MIC = 5 mg/mL, MBC = 10 mg/mL). According to the scientific literature, the microbial sensitivity of *Listeria monocytogenes*, *Pseudomonas aeruginosa*, *Salmonella typhimurium*, and *Escherichia coli* towards the alcoholic extracts of basil, summer savory, and tarragon are associated with the content of total phenolic compounds from the plant extracts, which act as antioxidants and cytoplasmic membrane destabilizing agents that lead to the bacterial cell death [26,27,28,29].

### 2.2. Plant Extracts Characterization

The values of the quality indices of the sausages with the addition of lyophilized extracts of basil, summer savory and tarragon were evaluated in a comparison with the control sample (sausages obtained by the classic recipe), Table 2.

According to the organoleptic analysis within the organized tastings, the values of the appreciation score decrease compared to the value given to the control sample, the highest values were recorded for samples with the addition of summer savory 0.05–4.99% points, the addition of basil 0.1–4.95% points, and the addition of tarragon 0.1–4.89% compared to the control sample, which was assessed by 5.00 points according to the standard pre-assessment system. Higher concentrations of lyophilized additives from the above-mentioned plants are felt stronger organoleptically, which depreciates the value of a meat product.

According to the data presented in Table 2, the moisture content of the sausage samples corresponds to the regulated permitted values [19]. It was found that samples with the addition of plant extracts are characterized by a higher moisture content. In the control sample, the moisture content was 64.37%, and in the samples with extracts, the increase in the value of this indicator was influenced by the concentration of the added extract. The samples with the highest extract concentration had the highest moisture content, thus SBE (0.3%)–69.16%; SSE (0.2%)–71.48% and SET (0.3%)–69.64%. This phenomenon can be explained by the fact that lyophilized extracts contain polysaccharides, which have the ability to bind and retain water in sausages, thus preventing its removal in heat treatment. During storage under refrigeration conditions, the moisture content was reduced in all the samples analysed. This gradual decrease in moisture content during storage was due to the loss by evaporation through the packaging material. Similar results have been reported by Heena Sharma et al. (2017) in chicken sausages incorporated with holy basil, cloves, and cassia essential oil [30].

The pH of the sausage samples was influenced by the addition of plant extracts, Table 2. The concentrations of the added plant extracts were found to lead to the decrease in the pH values of the sausages. The lowest pH value was determined in the samples with the highest extract concentration. Thus, in the SBE 0.3% was 6.12; SSE 0.2–6.10% and in STE 0.3–6.12%, while the pH of the control sample was 6.38. During storage, the pH value increases in all the researched samples. We explain an increase in pH in supplemented sausages by influence of redox-transformations of phenolic compounds from *Ocimum basilicum* L., and *Satureja hortensis* L. extracts, in which they are very rich [30,31,32,33].

The complex mechanism of microbial suppression under the influence of phenols includes the participation of phenols in various redox processes [34,35,36]. The main version of these is that in which phenol, initially weak acid, is transformed to neutral quinone [36,37]. This process can be described by the following general equation:

(1) $$-\mathrm{OH}\;\overset{-e^{-};-H^{+}}{\to }=O$$

Factors affecting the leaching of matrix compounds may include the polarity of the medium used. The action of polyphenolic compounds in the matrix of meat products is stabilized by the nonpolar compositions. Polar environments, such as water, allow changes in the levels of phenolic compounds. In contrast, if the environment is non-polar, the loss of compounds is lower due to the lack of diffusion or migration to the environment [38].

The addition of basil, summer savory and tarragon extracts in different concentrations leads to a non-essential decrease in water activity (a_w_), Table 2. In the case of basil samples, the a_w_ was not influenced by the increase in the concentration of added extracts, and in the case of summer savory samples, and tarragon, the value of this indicator varies from 0.876 c.u. up to 0.874 c.u. and from 0.874 c.u. up to 0.868 c.u. respectively. During storage, samples with extracts are characterized by values higher than a_w_. These results are correlated with the data obtained for the moisture content of the sausages, which is higher in samples with the addition of extracts.

Water activity and pH are important physicochemical indicators that determine whether a sausage sample will condition the growth of pathogenic microorganisms [39]. The growth of microorganisms was certified both in the control sample and in the sausage samples with the addition of basil, summer savory and tarragon. The experimental data in Table 3 represent the growth rate of the identified microorganisms after 24 h, 96 h (4 days) and 168 h (7 days) after manufacture.

After 24 h from manufacture all samples corresponded to microbiological standard indicators; coliforms bacteria, *Staphylococcus aureus* or *Salmonella* spp. we not detected.

After 96 h (4 days) from manufacture in the control sample and the one with 0.3% tarragon content and a number of 10^4^ CFU were identified; this exceeds the normative requirements. Coliforms bacteria, *Staphylococcus aureus* or *Salmonella* spp. were not detected.

After 7 days (168 h) from manufacture, in all samples a number of CFUs were identified that exceeded the normative requirements, except for the sample with 0.2% basil [39]. Coliform bacteria, *Staphylococcus aureus* or *Salmonella* spp. were not detected.

Sausages previously infected with standard strains *Staphylococcus aureus* ATCC 25923 and *Escherichia coli* ATCC 25922 were investigated for the growth rate of pathogenic microorganisms within 24 h and 48 h.

According to the obtained results, a higher number of colonies is observed in the control sample compared to the samples containing extracts. In the SBE samples less number of colonies for *Staphylococcus aureus* was observed after 48 h, and for Gram-negative bacilli the basil extracts showed a higher activity after 24 h. Basil extracts in concentrations of 0.1% and 0.3% were more active on *Staphylococcus aureus*, but concentrations of 0.2% and 0.3% had activity on Gram-negative bacilli. Summer savory and tarragon extracts had a more pronounced activity after 24 h on all species of bacteria tested. The summer savory extract in concentrations of 0.1% and tarragon extract in concentrations of 0.2% and 0.3% showed a more pronounced antimicrobial activity (Table 4). Probably, the microbial development in sausages, kept cold, is influenced not only by quality physicochemical indicators such as moisture content, water activity and pH, but also by the antimicrobial activity of the added extracts [40].

In the control sample it was determined more colonies of microorganisms compared to the other samples. In the samples of sausages with summer savory extract there is a diminished growth of *Staphylococcus aureus* strain, especially in the sample with a concentration of 0.2% summer savory. In the samples of sausages SBE 0.2% and SSE 0.2%, a smaller number of colonies of the genus *Escherichia coli* were observed.

There is a growing number of evidence that flavonoids have antibacterial activity against both Gram-positive and Gram-negative bacteria. The mechanisms of action of phenolic compounds on the bacterial cell have been partly attributed to damage the bacterial membrane, inhibition of virulence factors such as enzymes and toxins, and suppression of bacterial biofilm formation [41].

When evaluating the antimicrobial effect of different quantities of *Satureja montana* L. essential oil against *Clostridium perfringens* type A inoculated in mortadella-type sausages with different concentrations of sodium nitrite stored at 25 °C for 30 days, the population of target microorganisms was reduced compared to control samples [31]. In order to obtain safe products, with the use of natural additives the basil essential oil was added in the Italian-type sausage which showed a positive influence on reducing the count of *Staphylococcus aureus* until the 14th day of storage [40]. The addition of *Juniperus communis* L. essential oil at concentrations of 0.01, 0.05 and 0.10 μL/g to dry fermented sausage resulted in satisfying physico-chemical properties and improved oxidative stability [33].

## 3. Materials and Methods

### 3.1. Hydroalcoholic Extract Preparation

The plant material used in this study, basil (Kamis-Condimente SRL, Bucuresti, Romania), summer savory (Kamis-Condimente SRL, Bucuresti, Romania) and tarragon (Kamis-Condimente SRL, Bucuresti, Romania), was purchased from the local supermarket.

The powder obtained after plant pulverization with a grinder, was subjected to ultrasonic-assisted extraction with 80% ethanol, the ratio between plant and extraction solvent being 1:5 (*w*/*v*). The extractions were performed in a Transsonic T 310 ultrasonic bath, at room temperature for 30 min. The obtained extracts were centrifuged and stored at 4 °C until analysis. All the extracts were performed in triplicate.

In order to incorporate the extracts into the sausages, they were concentrated up to 10%, to remove the alcohol and then lyophilized.

### 3.2. Characterization of the Obtained Extracts

#### 3.2.1. Total Phenolic Content

An T80 UV-VIS spectrophotometer (PG Instruments Limited) was used to determine the total polyphenol content of the obtained extracts, using the Folin-Ciocâlteu method [42]. This method is based on the chemical reduction of the Folin-Ciocâlteu reagent, the resulting products giving a blue colored compound which is represented by a wide absorption band with a maximum of 765 nm. Thus, in a volumetric flask (10 mL) containing 5 mL of double-distilled water, 10 μL of extract and 0.5 mL of Folin-Ciocâlteu reagent were added. The obtained mixture was left to stand for 3 min, after which 1.5 mL of Na_2_CO_3_ (5 g/L) and bidistilled water up to 10 mL were added. The samples were stored at 50 °C (in a water bath) for 16 min in closed flasks, after that these were cooled to room temperature and their absorbance was read relative to the blank sample (double-distilled water). The total phenolic content was expressed as gallic acid equivalents (GAE) per 1 g of dried weight (DW) of plant.

#### 3.2.2. Antioxidant Capacity

The antioxidant capacity of the obtained extracts was determined by DPPH method (2,2′-diphenyl-picrylhydrazyl), following a slightly modified procedure reported by Brand-Williams et al. [43].

In order to determine the antioxidant capacity, a volume of 0.01 mL extract was added to 3.9 mL of DPPH radical solution (0.0025 g/100 mL of methanol). The resulting mixture was kept in the dark for 10 min, after which the absorbance of the mixture was measured at 515 nm to the control sample (0.01 mL extract added to 3.9 mL methanol). The results were expressed in mM Trolox/g dry plant material.

#### 3.2.3. Antimicrobial Activity

For the bioassay four bacterial strains *Listeria monocytogenes* (ATCC 19114), *Pseudomonas aeruginosa* (ATCC 27853), *Salmonella typhimurium* (ATCC 14028) and *Escherichia coli* (ATCC 25922) were taken into -account for this study. All of the tested microorganisms were obtained from Food Biotechnology Laboratory, Life Sciences Institute, University of Agricultural Sciences and Veterinary Medicine Cluj Napoca, Romania. For the antimicrobial activity evaluation, the obtained extract was evaporated to dryness under reduced pressure at 30 °C and re-suspended in 5 mL of bidistilled water.

Evaluation of the antimicrobial activity was done according to the guidelines of the Clinical Laboratory Standards Institute (CLSI) [44] using the standard broth microdilution technique, with few modifications. The bacteria were cultured on Muller-Hinton Agar and cultures were stored at 4 °C and subcultured once a month. The medium used for susceptibility testing was Tryptic Soy Broth (TSB) medium. Before antibacterial susceptibility testing, each aerobic bacteria was cultured overnight at 37 °C on Tryptic Soy Broth (TSB) medium. The bacterial cell suspensions were adjusted with sterile saline to a concentration of approximately 2 × 10^5^ CFU/mL in a final volume of 100 µL per well. The inoculum was stored at +4 °C for further use. Dilutions of the inoculums were cultured on solid Muller-Hinton (MH) to verify the absence of contamination of bacteria and to check the validity of the inoculums. Determinations of minimum inhibitory concentrations (MICs) were performed by a serial dilution technique using 96-well microtitre plates. The 100 µL Mueller–Hinton broth was placed into each of the 96 wells of the microplates. Aliquots of 100 µL of each ethanolic extract were added into the first rows of the microplates and twofold dilutions of the extracts were made by dispensing the solutions into the remaining wells. Afterwards, 10 μL of inoculum were added to all the wells. We used ethanol (40%) in water as a control. The microplates were incubated for 24–48 h at 37 °C. The MIC of the samples was detected following the addition of 20 μL (0.2 mg/mL) of resazurin solution to each well, and the plates were incubated 2 h at 37 °C. A change from blue to pink indicates reduction of resazurin and therefore bacterial growth. The MIC was defined as the lowest extract concentration that prevented this colour change, therefore inhibited the growth of the bacterial strain [45]. The minimum bactericidal concentrations (MBCs) were determined by serial subcultivation of a 2 μL into 96-microtitre plates containing 100 μL of MH broth per well and further incubation for 48 h at 37 °C. The lowest concentration of tested extract/compound/antibiotic with no visible bacteria growth was defined as MBC, indicating 99.5% killing of the original inoculum [45]. Streptomycin (Sigma P 7794, Santa Clara, CA, USA) (0.05–3 mg/mL) was used as positive control for bacterial growth. A 10% solution of ethanol in water was used as negative control.

### 3.3. Sausages Preparation

The sausage samples were prepared in laboratory conditions (semi-industrial) according to classic technology of manufacturing “Lacta” sausages, included in the group of boiled sausages. According to the recipe, expressed in kilograms of raw materials required to obtain 100 kg of the finished product (kg/100 kg), sausages were obtained from high quality beef (35 kg), semi-fat pork (48 kg), fatty pork (12 kg), powdered milk (2 kg), eggs (3 kg) is mixed with spices and auxiliary materials consisting of salt (1.87 kg), sugar (0.12 kg), ground black or white pepper (0.12 kg) and nutmeg (0.04 kg). Also, 25% ice was added to the sausage’s composition.

The control sample was obtained according to the classical unmodified technology. Subsequently, samples were prepared with lyophilized basil extracts 0.1%, 0.2%, 0.3% and summer savory 0.05%, 0.1%, 0.2% and tarragon 0.1%, 0.2%, 0.3%. The lyophilized extracts from the mentioned plants were rehydrated and added to the composition at the technological stage of cutterization and homogenization. The composition was mechanically stuffed into polyamide membrane. Sausages were cooked by steam at 83 ± 2 °C for 40 min to an internal temperature of 72 °C. After steam cooking, samples were immediately chilled with cold water shower. Finally, the sausages were stored at 4 ± 1 °C for 6 days.

### 3.4. Sausages Quality Analysis

The sausages were analyzed on the 1st, 3rd, and 6th days from production date in order to study the parameters’ evolution during storage. Quality indicators were determined by international standardized methods. The values of the quality indicators are regulated by legislation [39].

#### 3.4.1. Sensory Analysis of Sausages

Standard ISO 6658:2017 [46] was followed when performing the sensory analysis of the products. Exterior appearance, color in section, taste, odor, and consistency were assessed using the 5-point system by an expert panel of eleven trained food technologists (Staff of Department of Food Technology, Faculty of Food Technology, Technical University of Moldova). The 5-point assessment system includes the following scores: 5—very good; 4—good, 3—satisfactory, 2—poor, 1—bad. Table 5 shows the sensory characteristics of sausages.

#### 3.4.2. Moisture Content

The mass fraction of moisture was determined by drying in an oven (gravimetric method). The method is based on the weight loss of the sample to constant mass, due to the evaporation of water by heating in oven at 103 ± 2 °C at atmospheric pressure [47].

#### 3.4.3. pH Determination

The pH was determined by the express method using the Testo 205 pH meter (Testo Ltd., Alton, UK), used for determinations in semi-solid substances in food production and processing.

#### 3.4.4. Water Activity Determination

The determination of water activity (a_w_) was performed by the express method using the LabSwift-aw device (Novasina AG, Lachen, Switzerland), specially designed to determine the free water fraction in a test sample such as foods, cosmetics, or pharmaceuticals.

### 3.5. Microbiological Analysis of the Sausages

Sausages with the addition of lyophilized plant extracts were subjected to microbiological examination with the investigation of the total number of aerobic mesophilic microorganisms (TNGs), which provides data on the contamination degree of the product, coliforms bacteria which indicates the fecal contamination and pathogenic microorganism’s detection of the *Staphylococcus* and *Salmonella* genus. The microbiological indicators were determined by international standardized methods.

#### 3.5.1. TNG Determination

The method consists of the determination of the mesophilic aerobic organotrophic bacteria and it is based on the fact that the microbial cells present in the test sample, in contact with the solidified peptone agar, will each form visible colonies, after incubation at 30 °C for 48–72 h. It is also known as TNG (total number of germs). Taking into account the decimal dilution used and the number of colonies forming units (CFU), the number of microorganisms per gram produced is determined. This test was performed in accordance with SM EN ISO 4833-1 [48].

#### 3.5.2. Coliforms Bacteria Determination

Coliforms bacteria are bacteria that grow/develop in the specific temperature range of 35–37 °C, ferment lactose, with gas release when the analysis is performed on Coliforme MacConkey agar (lactose media) and under the conditions provided in the method specified according to SM ISO 4831/2006 [49].

#### 3.5.3. Staphylococcus Genus Determination

From the prepared sample, from the 10^−1^ dilution is taken 10 mL (it corresponds to 1 g of the inoculated samples) and added to the Giolitti–Cantoni Broth—the enrichment medium—and incubated for 24 h at 37 °C. After 24–48 h, the sample (Giolitti–Cantoni Broth) is inoculated on solid nutrient medium Baird Parker and is thermostated for 48 h at 37 °C. If there appears typical black colonies of 1.5–2.5 mm in diameter, with a lecithinase zone, then these colony are confirmed by a plasmacoagulase test.

The method of the *Staphylococcus* genus determination is based on the properties of the mannitol use under anaerobic conditions to produce the coagulase. *Staphylococcus* occurs in clusters formed by the cleavage of 0.8 μm diameter spherical cells. It forms smooth, slightly convex, glossy colonies with a creamy consistency and regular edges. Colony pigmentation varies from white to golden yellow depending on the species. In liquid media they produce turbidity, an annular film and, over time, a dusty deposit and clarification of the liquid. They are facultatively anaerobic bacteria that grow to an optimal temperature of 30–37 °C with an optimal pH of 7–7.5. Certain differential characters are used to diagnose species. *S. aureus*, unlike other Staphylococcus, produces phospholipoprotein lipase and can use egg yolks (lipolytic properties). It is tolerant to salt, lithium chloride, potassium thiocyanate, sodium azide, glycine, and polymyxin [49,50].

#### 3.5.4. Determination of Genus Salmonella

Bacteria from genus *Salmonella* belong to Gram-negative rods, mobile, with peritrich cilia, facultative aerobic or anaerobic, which can multiply in culture media and in food and produces endotoxins. Normally, the number of bacteria belonging to the *Salmonella* genus in food is absent, these being associated with a numerous microflora in which enterobacteria is usually predominant.

In order to create favourable conditions for the *Salmonella* genus, to be detected in products, the microbiological analysis involves several steps consisting of inoculation into liquid nutrients without selectivity, inoculation into nutrients with different degrees of selectivity to promote *Salmonella* multiplication, inoculation on selective media and differentiation media for the isolation of colonies characteristic for the *Salmonella* genus and confirmation by biochemical and serological tests of species belonging to the *Salmonella* genus. The detection method of the *Salmonella* genus is provided in the standard SM EN ISO 6579-1: 2017 [51].

### 3.6. Microbiological Analysis of the Infected (Contaminated) Sausages with Reference Strains

The antibacterial activity of the basil, summer savory and tarragon extracts added to the sausages was determined in situ. The control sample and the samples with the addition of lyophilized plant extracts were previously infected with bacterial strains: *Staphylococcus aureus* and *Escherichia coli*.

1.0 g of each sample was ground in a porcelain mortar and then 1 µL of the bacterial cultures standardized according to the McFarland opalescence optical standard of turbidity (0.5) was added. Infected samples containing various plant extracts were prepared and incubated in thermostat at 37 °C for 24 and 48 h in order to determine the antibacterial effect of extracts. 

On the second day, decimal dilutions of each infected sample were made. Thus, to each 1 g of the infected sample, 9 mL of saline solution (0.85% NaCl) was added. Six dilutions were done, after which from the dilutions -3 and -6, 2 drops were inoculated on appropriate media for the tested strains. The samples were incubated at 37 °C for 24 h [52,53].

### 3.7. Statistical Analyses

All calculations were done using Microsoft Office Excel 2007 (Microsoft, Redmond, WA, USA) and graphs were performed using ORIGIN8 (OriginLab Corporation, Northampton, MA, USA). Data obtained in this study are presented as mean values ± the standard error of the mean calculated from 3 parallel experiments. The comparison of average values was based on the one-way analysis of variance (ANOVA) according to Tukey’s test at significance level *p* ≤ 0.05, using Minitab 17 programme (Minitab Ltd., Coventry, UK).

## 4. Conclusions

Plant extracts can be used as an ingredient or as a packaging component for short or long storage periods (such as for fresh meat and sausages). Finally, the search for effective and practicable solutions for the implementation of these extracts in active packaging is advisable and could find immense interest in the future.

As a result of the tests performed, it was established that vegetable additives-lyophilized extracts of basil and summer savory in the recipe for the manufacture of “Lacta” sausages can control the growth rate of microorganisms, including pathogenic ones. This was determined by evaluating the multiplication of strains of microorganisms such as *Staphylococcus aureus* and *Escherichia coli*.

The TNMAFA in samples with plant additives in different concentrations is much lower compared to the control sample. Infected strains in these samples show a directed progressive growth and development of microorganism’s colonies.

Therefore, the use of vegetable additives in the recipe of meat products can mean two things: an improved nutritional value of the product and an increased shelf life of the product by keeping the microbiological risk under control.

Lyophilization is an advanced method that ensures the preservation of the biological value of products with a composition sensitive to high temperatures. By applying lyophilization on spice plants and plant extracts, the problem of the stability of bioactive compounds and subsequent application in food technologies in order to fortify food can be solved.

According to the organoleptic analysis within the organized tastings, the values of the appreciation score decreases compared to the value given to the control sample; the highest values were recorded for samples with the addition of summer savory 0.05–4.99% points, the addition of basil 0.1–4.95% points, and the addition of tarragon 0.1–4.89% compared to the control sample, which was assessed by 5.00 points according to the standard pre-assessment system. Higher concentrations of lyophilized additives from the above-mentioned plants are felt stronger organoleptically, which depreciates the value of a meat product.

## Figures and Tables

**Table 1:** Minimum inhibitory concentration (MIC) and minimum bactericidal concentration (MBC) of basil, summer savory, tarragon and streptomycin against bacterial strains tested with the microdilution method.

Bacterial Strain	Standard Antibiotic	MIC, mg/mL			MBC, mg/mL		
		Basil	Summer Savory	Tarragon	Basil	Summer Savory	Tarragon
*Listeria* *monocytogenes*	0.03	1.25	0.31	1.25	2.50	0.62	2.50
*Pseudomonas* *aeruginosa*	0.12	5.00	5.00	2.50	10.00	10.00	5.00
*Salmonella* *typhimurium*	0.12	5.00	5.00	5.00	10.00	10.00	10.00
*Escherichia coli*	0.24	5.00	2.50	5.00	10.00	5.00	10.00

**Table 2:** Physicochemical quality indicators and sensory profile of sausages with added lyophilized extracts of basil, summer savory and tarragon compared to the control (the results are presented as means ± standard deviation).

Quality Indicators	Storage Time	Control	SBE 0.1%	SBE 0.2%	SBE 0.3%	SSE 0.05%	SSE 0.1%	SSE 0.2%	STE 0.1%	STE 0.2%	STE 0.3%
Moisture content, %	1st day	64.37 ± 0.09 _f_	67.38 ± 0.06 _e_	69.07 ± 0.06 _d_	69.16 ± 0.15 _c,d_	69.33 ± 0.09 _b,c,d_	69.44 ± 0.06 _b,c_	71.48 ± 0.09 _a_	69.48 ± 0.12 _b,c_	69.56 ± 0.03 _b_	69.64 ± 0.06 _b_
	3rd day	58.1 ± 0.15 _g_	59.3 ± 0.03 _f_	59.99 ± 0.03 _e_	61.06 ± 0.09 _c,d_	59.32 ± 0.15 _f_	60.28 ± 0.06 _e_	60.78 ± 0.03 _d_	62.51 ± 0.09 _a_	62.16 ± 0.06 _b_	61.09 ± 0.03 _c_
	6th day	54.89 ± 0.15 _d_	55.52 ± 0.06 _c_	55.57 ± 0.06 _c_	55.94 ± 0.12 _b_	55.04 ± 0.03 _d_	55.11 ± 0.09 _d_	55.83 ± 0.03 _b,c_	55.74 ± 0.12 _b,c_	56.41 ± 0.03 _a_	55.55 ± 0.06 _c_
Active acidity pH	1st day	6.38 ± 0.06 _a_	6.15 ± 0.12 _a_	6.13 ± 0.06 _a_	6.12 ± 0.06 _a_	6.16 ± 0.09 _a_	6.12 ± 0.03 _a_	6.10 ± 0.09 _a_	6.29 ± 0.12 _a_	6.14 ± 0.03 _a_	6.12 ± 0.06 _a_
	3rd day	6.40 ± 0.06 _a_	6.17 ± 0.09 _a_	6.26 ± 0.06 _a_	6.22 ± 0.09 _a_	6.33 ± 0.03 _a_	6.24 ± 0.12 _a_	6.28 ± 0.12 _a_	6.41 ± 0.12 _a_	6.33 ± 0.09 _a_	6.28 ± 0.03 _a_
	6th day	6.32 ± 0.09 _a_	6.24 ± 0.06 _a_	6.34 ± 0.12 _a_	6.32 ± 0.06 _a_	6.30 ± 0.09 _a_	6.24 ± 0.12 _a_	6.24 ± 0.12 _a_	6.28 ± 0.12 _a_	6.35 ± 0.06 _a_	6.26 ± 0.12 _a_
Water activity a_w_, c.u.	1st day	0.875 ± 0.003 _a_	0.873 ± 0.003 _a_	0.873 ± 0.003 _a_	0.873 ± 0.003 _a_	0.876 ± 0.003 _a_	0.875 ± 0.002 _a_	0.874 ± 0.000 _a_	0.874 ± 0.000 _a_	0.871 ± 0.003 _a_	0.868 ± 0.003 _a_
	3rd day	0.869 ± 0.000 _a_	0.871 ± 0.003 _a_	0.871 ± 0.000 _a_	0.872 ± 0.000 _a_	0.871 ± 0.000 _a_	0.871 ± 0.003 _a_	0.869 ± 0.003 _a_	0.874 ± 0.000 _a_	0.871 ± 0.003 _a_	0.868 ± 0.003 _a_
	6th day	0.866 ± 0.000 _a_	0.869 ± 0.000 _a_	0.869 ± 0.003 _a_	0.870 ± 0.003 _a_	0.869 ± 0.003 _a_	0.869 ± 0.000 _a_	0.869 ± 0.002 _a_	0.874 ± 0.003 _a_	0.871 ± 0.003 _a_	0.868 ± 0.003 _a_
Average score of sensory profile	3rd day	5.00 ± 0.00 _a_	4.95 ± 0.02 _a,b_	4.68 ± 0.03 _c_	4.32 ± 0.04 _e_	4.99 ± 0.01 _a,b_	4.66 ± 0.03 _c_	4.35 ± 0.03 _e_	4.89 ± 0.01 _b_	4.51 ± 0.03 _d_	3.99 ± 0.04 _f_
Exterior appearance		5.00 ± 0.00 _a_	5.00 ± 0.00 _a_	4.95 ± 0.01 _a,b_	4.90 ± 0.03 _b,c_	5.00 ± 0.00 _a_	4.98 ± 0.01 _a_	4.94 ± 0.02 _a,b,c_	5.00 ± 0.00 _a_	4.93 ± 0.02 _a,b,c_	4.87 ± 0.05 _c_
Color and appearance in section		5.00 ± 0.00 _a_	5.00 ± 0.00 _a_	4.75 ± 0.02 _b_	4.45 ± 0.01 _d_	5.00 ± 0.00 _a_	4.75 ± 0.02 _b_	4.60 ± 0.05 _c_	5.00 ± 0.00 _a_	4.50 ± 0.02 _c,d_	3.75 ± 0.06 _e_
Odor		5.00 ± 0.00 _a_	5.00 ± 0.00 _a_	4.87 ± 0.04 _b_	3.83 ± 0.06 _f_	5.00 ± 0.00 _a_	4.37 ± 0.02 _d_	4.00 ± 0.01 _e_	4.75 ± 0.02 _c_	3.83 ± 0.04 _f_	3.63 ± 0.02 _g_
Taste		5.00 ± 0.00 _a_	4.75 ± 0.11 _b_	3.83 ± 0.05 _d_	3.46 ± 0.07 _e_	4.95 ± 0.02 _a_	4.27 ± 0.08 _c_	3.35 ± 0.10 _e,f_	4.70 ± 0.10 _b_	4.33 ± 0.05 _c_	3.25 ± 0.05 _f_
Cosistency		5.00 ± 0.00 _a_	4.99 ± 0.01 _a_	4.98 ± 0.02 _a,b_	4.98 ± 0.01 _a,b_	4.99 ± 0.01 _a,b_	4.95 ± 0.02 _a,b_	4.87 ± 0.07 _b_	4.99 ± 0.01 _a_	4.98 ± 0.02 _a,b_	4.45 ± 0.05 _c_

Different letters (_a–g_) designate statistically different results determined by Tukey’s test (*p* < 0.05). SBE-sausages with basil extract; SSE-sausages with summer savory extract; STE-sausages with tarragon extract.

**Table 3:** The identified microorganisms after a period of time since the manufacture of sausages.

Sample/Storage Time	TNMAFA, CFU/g	Coliforms Bacteriain 1 g	*Staphylococcus aureus* in 1 g	*Salmonella* spp. in 25 g
*After 24 h*				
Control	10^3^	-	-	-
SBE 0.1%	10^3^	-	-	-
SBE 0.2%	10^3^	-	-	-
SBE 0.3%	10^3^	-	-	-
SSE 0.05%	10^3^	-	-	-
SSE 0.1%	10^2^	-	-	-
SSE 0.2%	10^3^	-	-	-
SET 0.1%	10^3^	-	-	-
SET 0.2%	10^3^	-	-	-
SET 0.3%	10^2^	-	-	-
*After 96 h (4 days)*				
Control	10^4^	-	-	-
SBE 0.1%	10^3^	-	-	-
SBE 0.2%	10^3^	-	-	-
SBE 0.3%	10^2^	-	-	-
SSE 0.05%	10^3^	-	-	-
SSE 0.1%	10^3^	-	-	-
SSE 0.2%	10^3^	-	-	-
SET 0.1%	10^2^	-	-	-
SET 0.2%	10^3^	-	-	-
SET 0.3%	10^4^	-	-	-
*After 168 h (7 days)*				
Control	10^5^	-	-	-
SBE 0.1%	10^4^	-	-	-
SBE 0.2%	10^3^	-	-	-
SBE 0.3%	10^4^	-	-	-
SSE 0.05%	10^4^	-	-	-
SSE 0.1%	10^4^	-	-	-
SSE 0.2%	10^4^	-	-	-
SET 0.1%	10^4^	-	-	-
SET 0.2%	10^4^	-	-	-
SET 0.3%	10^5^	-	-	-

TNMAFA–total number of mesophilic aerobic and facultative anaerobic bacteria; “-” absence of growth; CFU—colony forming unit. SBE—sausages with basil extract; SSE—sausages with summer savory; STE—sausages with tarragon.

**Table 4:** The results of in situ monitoring of growing pathogenic strains.

Sample	*Staphylococcus aureus* mln/g		*Escherichia coli* mln/g	
	24 h	48 h	24 h	48 h
Control	320	650	528	788
SBE 0.1%	312	26	67	232
SBE 0.2%	286	160	52	184
SBE 0.3%	242	74	46	242
SSE 0.05%	263	316	216	244
SSE 0.1%	146	136	104	103
SSE 0.2%	102	216	88	196
SET 0.1%	316	236	146	702
SET 0.2%	206	256	128	248
SET 0.3%	112	221	106	256

SBE—sausages with basil extract; SSE—sausages with summer savory extract; STE—sausages with tarragon extract.

**Table 5:** The sensory characteristics for the evaluation of sausages.

Sensory Characteristic	Product Description
Exterior appearance	Small sticks with a clean, dry surface, without stains, adhesions, affluences of composition and ruptures of the membrane. The ends of the membranes of small bars are twisted or tied with string or thread.
Color and appearance in section	The color of the composition from light pink (control) to the color characteristic of the type of plant extract, finely chopped with pieces of food ingredients other than meat, according to the recipe, mixed evenly and without gaps. The presence of fine porosity in the form of gaps and the insignificant presence of coarse connective tissue are allowed.
Odor	Characteristic of the type of product with an odor of respective plant extract, without a foreign odor.
Taste	Characteristic of the type of product with a taste of respective plant extract, without foreign taste.
Consistency	Fine, juicy (hot)

## Data Availability

Not applicable.

## Sample Availability

Samples of the compounds are not available from the authors.
